# Osteoconductive resorption characteristics of a novel biocomposite suture anchor material in rotator cuff repair

**DOI:** 10.1186/s13018-018-1049-x

**Published:** 2019-01-09

**Authors:** Jan Vonhoegen, Dominik John, Constanze Hägermann

**Affiliations:** Klinik Am Ring, Cologne, Germany

**Keywords:** Arthroscopic rotator cuff repair, Suture anchor, Bioabsorbable, PGLA, Osteolysis

## Abstract

**Background:**

Bioabsorbable suture anchors have been associated with bone-derived complications, such as osteolysis and cyst formation, after rotator cuff repair. The purpose of this study was to assess the osseous degradation process of the novel biocomposite suture anchor material polylactic-co-glycolic acid (PLGA)/beta-tricalcium phosphate (ß-TCP)/calcium sulfate (CS) after arthroscopic single-row rotator cuff repair. The focus of interest was the appearance of osteolysis and the rate of total resorption of the implants after 21 months.

**Methods:**

Forty-eight patients with 82 implanted suture anchors who had undergone arthroscopic rotator cuff repair between January 2015 and March 2016 at our institution were retrospectively evaluated by postoperative magnetic resonance imaging. The appearance of osteolysis was classified by measurement of the peri-implant fluid. The degree of resorption was measured by grading the persistent visibility of the anchor structures. The integrity of the rotator cuff tendon was analyzed to discover possible retear or anchor pull-out complications.

**Results:**

After a follow-up of 21.2 (± 5.4) months, osteolysis was detected in only two anchors (2.4%), and none of these defects exceeded the diameter of the former suture anchor (5.5 mm). Fifty percent of the anchors were fully degraded and no longer visible. Furthermore, only two retears of the rotator cuff occurred, and no anchor pull-out complications were detected.

**Conclusion:**

PGLA/β-TCP/CS is a fully resorbable and osteoconductive suture anchor material that seems to have superior resorption characteristics compared to those of other bioabsorbable suture anchor materials commonly used in arthroscopic rotator cuff repair.

**Trial registration:**

The presented study was retrospectively registered by the commission for ethics at the Ärztekammer Nordrhein with the registration number 2016433 on January 17, 2017. All participating patients gave written consent for participation and the publication of their data.

**Level of evidence:**

IV

## Introduction

In the past, the use of suture anchors in arthroscopic rotator cuff repair (RCR) was often associated with complications depending on the design and the anchor materials used [1, 2]. The first commonly used suture anchors, made of titanium, compromised postoperative controls with magnetic resonance imaging (MRI) by causing artifacts, and in cases of revision surgery, the persistent anchor material limited the positioning of new suture anchors [3]. The first generation of bioabsorbable anchor materials showed an increased number of bone cysts and osteolysis. Additionally, a higher number of dislocated anchors was reported to cause persistent pain, postoperative frozen shoulder, and cartilage damage [4–6]. The initial bioabsorbable anchors were made out of poly-glycolic acid (PGA) and showed rapid absorption, resulting in a loss of primary stability at approximately 4 weeks after implantation and RCR [7]. The problem of rapid absorption was solved by replacing PGA and using poly-l-lactate (PLLA) [8] or a combination of l-lactate and d-lactate (PLDLA). However, these anchor materials with lower absorption rates were visible in MRI investigations up to 7 years postoperatively, and osteolysis and peri-implant cyst formations in the anchor area were detectable [9, 10]. In conclusion, perfect suitable bioresorbable suture anchor material has not yet been identified. In this study, we investigated the resorption characteristics of the novel biocomposite suture anchor material Regenesorb® to close the gap between primary stability and absorption.

The novel biocomposite suture anchor material used in this study consists of 65% poly-lactic-co-glycolic acid (PLGA)/15% beta-tricalcium phosphate (ß-TCP) and 20% calcium sulfate (CS). The Regenesorb® suture anchors contain an open helix structure to facilitate the osteoconductive infiltration of new bone material. Such an osteoconductive effect was described for the two-component material PLGA/ß-TCP in a review analysis of 668 patients. An average resorption time of 36 months was reported in this review [11]. However, the review did not include any studies with the abovementioned combination of three materials and did not consider the specialties in rotator cuff repair. To validate whether the three-component material has superior resorption and osteoconductive characteristics compared to those of other commonly used bioabsorbable materials in arthroscopic rotator cuff repair in the present study, 48 patients with 82 suture anchors were retrospectively evaluated by postoperative magnetic resonance imaging (MRI). The appearance of bone cysts and osteolysis was analyzed in all cases.

Two hypotheses were clarified in this study:

- The new suture anchor material will be completely reabsorbed within 2 years after arthroscopic rotator cuff repair, without the formation of bone cysts and/or osteolysis and will be replaced by new bone formation.
- The used anchor material will provide sufficient primary stability 2 years postoperatively and will lead to the complete healing of the tendon after rotator cuff refixation.

## Materials and methods

### Inclusion and exclusion criteria

All patients underwent MRI examinations preoperatively. The tears of the rotator cuff were classified according to Patte [12] and Bateman [13]. Only patients with a Patte I or II rupture were included. The MRI findings were confirmed intraoperatively and classified according to Bayne and Bateman [13]. Patients with concomitant diseases, such as acromioclavicular joint arthritis, tendinitis of the long head of the biceps tendon, and subacromial bursitis, were included. Patients undergoing previous shoulder surgeries and revision rotator cuff repairs were excluded as well as patients with known co-existing osteoporotic diseases and frozen shoulder. The ethical review board of the Medical Association of North Rhine (Ärztekammer Nordrhein) approved this study. All participants gave written informed consent.

### Surgical procedure

All procedures were performed by two surgeons at our institution under general anesthesia with the patient placed in the beach chair position. Preoperative intravenous antibiotics were administered. A standardized diagnostic arthroscopy was performed via an anterior and posterior portal to confirm the diagnosis and inspect the glenohumeral joint. Depending on the concomitant pathologies found during diagnostic arthroscopy, a tenotomy of the long head of the biceps tendon was performed if necessary. A standard lateral portal was established to visualize and classify the tear size according to Bateman and Patte [12, 13]. Subacromial bursectomy and a lateral acromioplasty were performed in every case. Lateral resection of the clavicle was performed in cases of symptomatic acromioclavicular joint arthritis. If needed, an additional anterior lateral portal was established for optimal anchor positioning. The torn part of the tendon and the rotator cuff footprint were debrided using a shaver and a radiofrequency device (Whirlwind, Fa. Smith & Nephew, Hamburg, Germany). Tendon mobilization was performed if necessary. The cortical bone part of the greater tuberosity was not decompressed. Prior to implantation of the suture anchors, the bone was prepared using a 5.5-mm threaded dilator without impaction, and the anchors were placed through the lateral portal (one to three anchors depending on the tear size). The rotator cuff was reconstructed using 5.5 mm Healicoil Regenesorb® (Smith & Nephew GmbH, Hamburg, Germany) suture anchors with a modified Mason-Allen stitch technique. The integrity of the repair was assessed from the bursal and articular sides.

### Suture anchor material

The Healicoil Regenesorb® anchor comprises a co-polymer consisting of 65% PLGA (poly-l-lactic co-glycolic acid), 15% β-tricalcium phosphate, and 20% calcium sulfate. The maximum diameter of the anchor is 5.5 mm; the length is 18.5 mm. The three components PLGA, β-TCP, and CS have different intraosseous resorption characteristics, as shown in animal models. PLGA, a co-polymer out of PLLA and PGA (ratio, 85:15), showed a resorption time of 24 months [14]. The other two components have reported shorter resorption times (β-TCP 18 months and CS 4–12 weeks) [15, 16].

The combination of the three components should guarantee primary stability for 6 months according to the manufacturer’s information (Smith & Nephew Reports: 15001873, 15002036). Beta-TCP and CS have been reported to have osteoconductive properties to facilitate bone infiltration [17]. In addition, the open helix structure of the anchor also supports fast bone infiltration [17; Smith & Nephew Reports: 15000897, 15001194, 15000921, 15000919].

### Rehabilitation

Postoperatively, the arm was placed in a shoulder brace (medi Arm Fix, medi GmbH & Co. KG; Bayreuth, Germany) without an abduction pillow for 6 weeks. During the first 4 weeks, only passive mobilization of the affected shoulder was permitted. Active forward flexion and abduction up to 45° were allowed after 5 weeks. The passive and active range of motion was increased gradually past the fifth week postoperatively.

### Radiological image evaluation

All MRI examinations were performed according to a standardized algorithm employing a 3.0-T (Magnetom Trio/Verio Siemens Medical Solutions, Erlangen, Germany) magnetic resonance scanner with a dedicated shoulder coil. The patients were placed in a supine position with the forearm in a neutral position.

The following protocol was used: axial fast spin-echo proton density-weighted image with fat saturation (repetition time (TR)/echo time (TE), 2300–3900/30–60 ms; slice thickness, 3 mm; slice gap, 0.3 mm; field of view (FOV), 14 cm; matrix, 384 × 269 pixels; echo train length, 7), oblique coronal and sagittal fast spin-echo T2-weighted images with fat saturation Fat-Sat (TR/TE, 2300–4600/30–50 ms; slice thickness, 3 mm; slice gap, 0 mm; FOV, 14 cm; matrix, 384 × 269 pixels; ETL, 12), and oblique coronal and extended oblique sagittal fast spin-echo T1-weighted images (TR/TE, 400–800/10–15 ms; slice thickness, 3–4 mm; slice gap, 0–0.4 mm; FOV, 14 cm for coronal and 16 cm for sagittal images; matrix, 384 × 269 pixels; ETL, 4). The program Examion X-AQS version 3.02.05 (Examion GmbH, Fellbach, Germany) was used for optimal picture display and analysis.

The following grading for the resorption of the installed suture anchors was used (Table 1):

**Table 1:** Resorption grades of the anchors [10]

Grading	Anchor resorption
Grade 1	Clearly visible
Grade 2	Visible
Grade 3	Barely visible, partially oedematous bleaching
Grade 4	Complete resorption

The fluid signal in the anchor area on T2-weighted MRI scans was graded as follows (Table 2):

**Table 2:** Grading of osteolysis reaction with 5.5 mm anchor diameter [18]

Grading	Osteolysis reaction
Grade 0	No fluid
Grade 1	< 1 mm diameter of fluid accumulation
Grade 2	1–5.5 mm diameter of fluid accumulation, definition osteolysis
Grade 3	> 5.5–6.5 mm diameter of fluid accumulation peri-implant
Grade 4	> 6.5 mm diameter of fluid accumulation, definition cyst

In addition, the healing process and the integrity of the reconstructed rotator cuff were analyzed according to the classification of Sugaya et al. [19].

### Statistical analyses

All statistical analyses were performed with Microsoft Excel for Mac (version 14.0.0). The size of the retear, grade of retraction, and osteolysis were determined with Turkey’s multiple comparison test and two-way analysis of variance (ANOVA). The data were analyzed with GraphPad Prism version 7 (GraphPad Software, CA, USA). Statistical significance was set at *p* < 0.05.

## Results

**Fig. 1:**
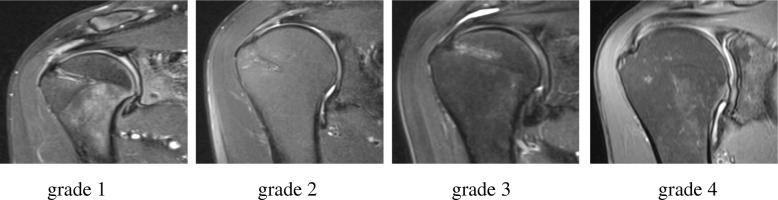
Grading of the resorption of the anchors. Grading system as described in Table 1. Coronal MRI-slices, fast spin-echo T2-weighted images with fat saturation Fat-Sat (TR/TE, 2300–4600/30–50 ms; slice thickness, 3 mm; slice gap, 0.3 mm)

Forty-eight patients [26 women (54%)/22 men (46%)] were included in this study. The average follow-up time was 21.2 (± 5.4) months. The determination of the intra-operative defect size showed 42.2% Bateman I and 57.8% Bateman II defects of the rotator cuff tendon. Altogether, 82 anchors were implanted, yielding an average of 1.71 (± 0.7; min, 1; max, 3) suture anchors per patient. The follow-up examinations were performed at 21.2 (± 5.4) months. A total of 50.0% (w, 27.1%; m, 22.9%) of patients showed complete resorption of the anchor material (Fig. 1, grade 4). Nearly complete resorption with some persistent residual material was observed in 25% (w, 16.7%; m, 8.3%) of patients (Fig. 1, grade 3). Nevertheless, a visible anchor structure with liquid signals within a helical structure (Fig. 1, grade 2) or a persistent clearly visible helicoidal anchor structure (Fig. 1, grade 1) was detected in 12.5% (w, 8.3%; m, 4.2%) of patients (Table 3).

**Table 3:** Anchor resorption grades and patients: grade 1, clearly visible structure; grade 2, visible structure; grade 3, hardly visible, partially oedematous bleaching; and grade 4, complete resorption

Anchor resorption grades by patients and anchors		
Grade	Patients	Anchors
1	6	9
2	6	8
3	12	23
4	24	42

There was no significant statistical correlation between anchor resorption and age, retear, defect size, gender, number of anchors, and grade of retraction (Table 4).

**Table 4:** Resorption grade correlated with age (> 60 years/< 60 years), retear, defect size, gender, and number of anchors and retraction grade (statistical analysis)

Resorption	Grade 1	Grade 2	Grade 3	Grade 4
Age (> 60 years/< 60 years)	> 0.9999 (ns)/0.9897 (ns)	0.6108 (ns)/> 0.9999 (ns)	> 0.999 (ns)/0.0197*	0.6108 (ns)/0.0089**
Retear	> 0.9999 (ns)	> 0.9999 (ns)	0.9262 (ns)	0.9262 (ns)
Defect size (Bateman 1 or 2/Bateman 3 or 4)	> 0.9999 (ns)/> 0.9999 (ns)	0.4267 (ns)/> 0.9999 (ns)	0.0197*/> 0.9999(ns)	0.0003***/> 0.9999 (ns)
Gender (m/w)	0.9897 (ns)/0.9897 (ns)	0.9252 (ns)/0.7921 (ns)	0.1550 (ns)/0.9262 (ns)	0.0830 (ns)/0.2680 (ns)
Number of anchors (1/2/3)	> 0.9999 (ns)/0.9897 (ns)/0.9897 (ns)	0.9897 (ns)/0.2680 (ns)/> 0.9999 (ns)	0.1550 (ns)/0.9897 (ns)/0.9262 (ns)	0.2680 (ns)/0.0830 (ns)/0.9897 (ns)
Retraction grade (Patte 1/2)	0.9897 (ns)/0.9897 (ns)/> 0.9999	0.9262 (ns)/0.7921 (ns)/> 0.9999 (ns)	0.4247 (ns)/0.4247 (ns)/> 0.9999 (ns)	0.0830 (ns)/0.1550 (ns)/> 0.9999 (ns)

* = *p* < 0,05, ** = *p* < 0,01, *** = *p* < 0,001

**Fig. 2:**
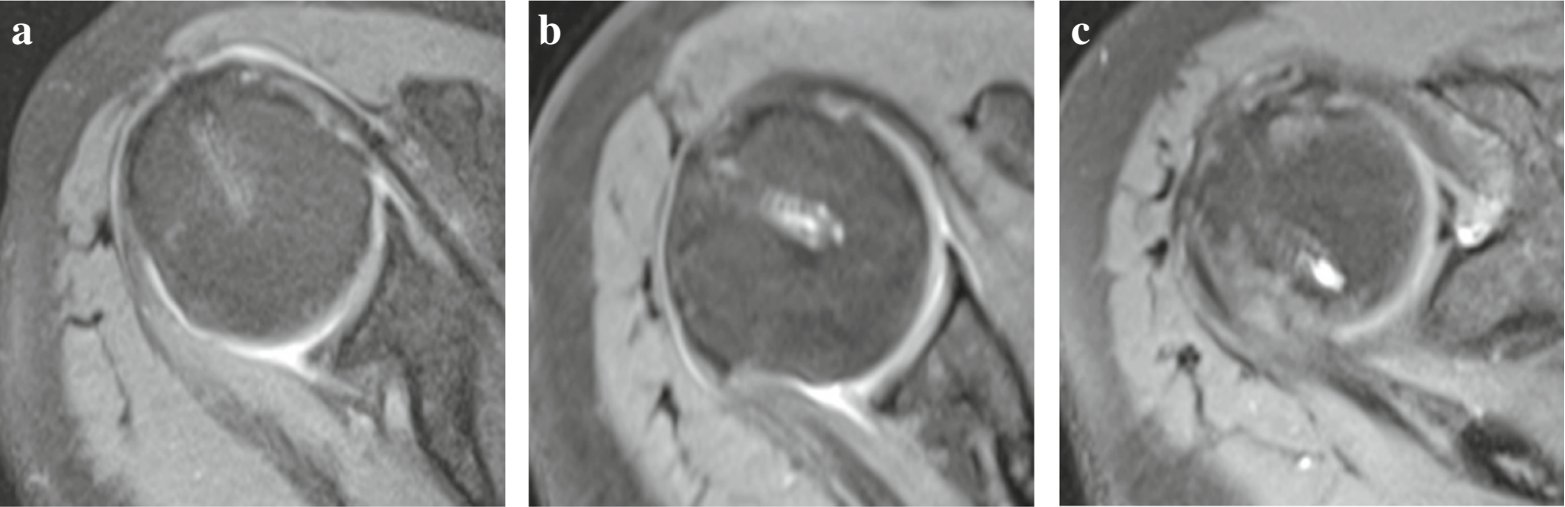
Grade of osteolysis. **a** Osteolysis grade 0 without fluid signal. **b** Osteolysis grade 1 with a punctual fluid signal within the anchor area. **c** Osteolysis grade 2 with separable sections of anchor material and fluid accumulation at the tip of the anchor

No fluid signal was found inside the helical structure in 69.5% of the anchors (Fig. 2a, grade 0). We observed a small focal fluid accumulation reaction of less than 1 mm diameter within the helical structure in 28.1% of the anchors (Fig. 2b, grade 1). Only 2 out 82 anchors (2.4%) led to osteolytic structures (Fig. 2c, grade 2) that were smaller (grade 2, 4.1 mm and 4.5 mm) than the original anchor diameter (5.5 mm).

An osteolytic reaction with a diameter greater than the anchor or perianchor cyst formation (osteolysis grade 3 or 4) was not observed in any patient (Table 5).

**Table 5:** Fluid detection classified by patient and anchors: grade 0, no fluid signal; grade 1, < 1 mm diameter of fluid accumulation; grade 2, 1–5.5 mm diameter of fluid accumulation, definitive osteolysis; grade 3, > 5.5 mm–6.5 mm diameter of fluid accumulation surrounding the implant; and grade 4, > 6.5 mm diameter of fluid accumulation around the former anchor, definitive cyst

Fluid collection grades by patients and anchors		
Grade	Patients	Anchors
0	33	57
1	13	23
2	2	2
3	0	0
4	0	0

There was no significant statistical correlation between osteolysis, age or gender of the patients, retear rate, or size of the defect (Table 6).

**Table 6:** Osteolysis in correlation with age (> 60 years/< 60 years), retear, defect size, gender, and number of anchors and grade of retraction (statistical analysis)

Osteolysis	Grade 0	Grade 1	Grade 2
Age (> 60 years/< 60 years)	0.4308 (ns)/0.9969 (ns)	0.9097 (ns)/0.3346 (ns)	> 0.9999 (ns)/0.9969 (ns)
Retear	0.9969 (ns)	> 0.9999 (ns)	> 0.9999 (ns)
Defect size (Bateman 1 or 2/Bateman 3 or 4)	0.0398*/> 0.9999 (ns)	0.3346 (ns) vs > 0.9999 (ns)	0.9969 (ns) vs > 0.9999 (ns)
Gender (m/w)	0.5364 (ns)/0.6449 (ns)	0.9853 (ns)/0.6449 (ns)	0.9969 (ns)/> 0.9999 (ns)
Number of anchors (1/2)	0.5364 (ns)/0.7484 (ns)/0.9998 (ns)	0.9580 (ns)/0.8389 (ns)/0.9998 (ns)	> 0.9999 (ns)/0.9969 (ns)/> 0.9999 (ns)
Retraction grade (Patte 1/2)	0.1835 (ns)/0.9580 (ns)/> 0.9999 (ns)	0.4308 (ns)/0.9998 (ns)/> 0.9999 (ns)	> 0.9999 (ns)/0.9969 (ns)/> 0.9999 (ns)

* = *p* < 0,05

**Fig. 3:**
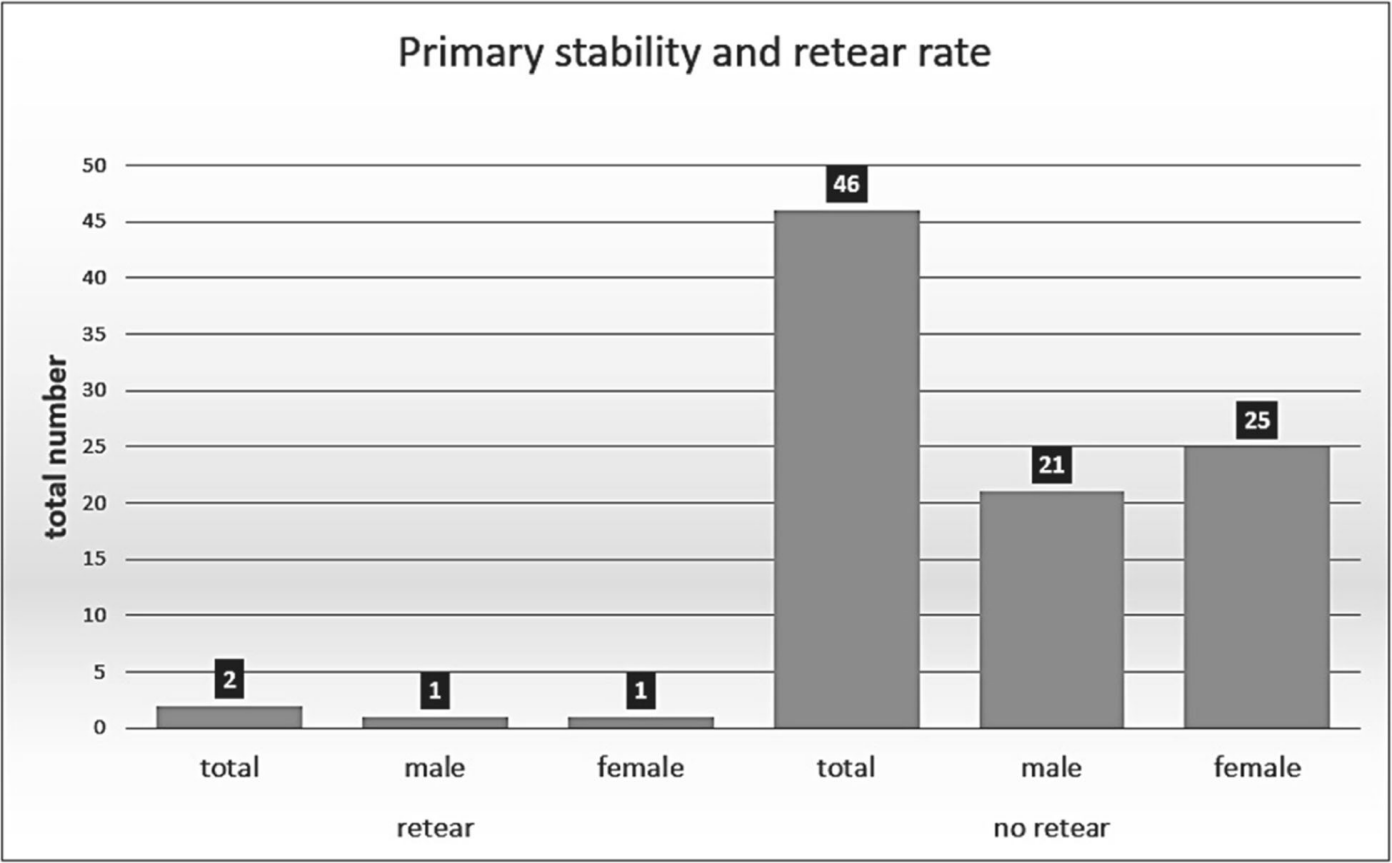
Distribution of primary stability and retear rate among gender

A retear of the rotator cuff tendon was found in two patients postoperatively (4.2%; m = 1/f = 1) (Fig. 3).

## Discussion

The present study demonstrates that the novel suture anchor material PLGA/β-TCP/CS is mostly absorbed at 21 months after RCR. At the time of the follow-up examinations, 75% of implants could not be distinguished from adjacent bone material by MRI examination using T2-weighted images. MRI imaging is superior compared to computer tomography analysis in detecting soft tissue damage and tendon retear [20]. Severe osteolysis and cyst formation (grades 3 and 4) were not observed at any of the 82 anchors. Furthermore, PLGA/β-TCP/CS showed sufficient primary stability for the 21-month observation period and led to the complete healing of the refixated supraspinatus tendon in 46 out of 48 (96%) patients.

Osteolysis and cyst formation severely deteriorate the situation in rotator cuff repair if revision surgery is needed [1]. Thus, the use of bioabsorbable anchor material with reliable degradation characteristics is highly desirable compared to metal implants, polyetheretherketone (PEEK) anchors or bioabsorbable anchors with an unpredictable, long degradation time. For example, PLLA anchor material was still detectable after an average time of 28 months postoperatively [10]. In contrast, resorption times similar to those of PLGA/β-TCP/CS were reported for PDLLA biointerference screw material after anterior cruciate ligament reconstruction [21].

Osteolytic structures (grade 2; smaller than the diameter of the anchor used) were observed for only two anchors (2.4%). There was no significant correlation between age, gender, or retears of the RCT and defect size. The significantly lower number of osteolysis and cyst formation using PLGA/β-TCP/CS anchors is an important difference compared to using PLLA anchors, for which different reports describe up to 70% osteolysis formation [10, 18]. In the study of Kim et al., the higher number of anchors used in the double-row technique may have resulted in an increase in the local intraosseous pressure, thereby reducing the blood flow in the tubercular majus and consequently may have led to an increase in cyst formation. Pilge et al. found 47% osteolysis for PDLDA anchors used in 30 patients with open rotator cuff repair [22]. Chung et al. observed osteolysis and partially cystic lesions in 60% of cases at 7 months after PLGA implants with a double-row technique [23]. Cyst formation was also reported when PEEK anchors were used [24, 10].

The findings described in the present study support the view that PLGA/β-TCP/CS is a good alternative to metal anchors and PEEK anchors in patients with good bone quality. Compared to PLLA and PDLDA, PLGA/β-TCP/CS seems to have superior characteristics regarding degradation time and the occurrence of osteolysis and cyst formation. A resorption time similar to that of PLGA/β-TCP/CS has been reported for PLGA and β-TCP. Barber et al. reported in a review study with 668 patients an 88% resorption rate at 28 months postoperatively. Newly generated bone was detectable in 63% of the former PLGA/β-TCP anchors [11]. Compared to the Regenesorb® suture anchors used in the present study, with new bone formation in 96% of the cases, Barber et al. showed the limited osteoconductive activity of PLGA/ β-TCP co-polymers. The addition of CS and the open helix structure of Regenesorb® anchors seem to facilitate bone infiltration. These osteoconductive characteristics of PLGA/ß-TCP/HA have been described previously for bioabsorbable interference screws with a much larger diameter [14]. The relatively smaller diameter of the Regenesorb® suture anchors compared to that of the interference screws used in anterior cruciate ligament reconstruction seems to enhance absorption and the formation of new bone [14].

The use of PLGA/β-TCP/CS suture anchors resulted in strong primary stability with good healing of the refixated rotator cuff tendon. Retear of the reconstructed tendon was observed in only two patients. There was no significant correlation between retear and osteolysis. Good bone quality at the rotator cuff footprint on the greater tuberosity is a requirement for adequate healing of the tendon [25]. It is advantageous to prevent cyst formation and fluid assembly in this area by using a suture anchor material with short and complication-free degradation characteristics.

This retrospective study with postoperative MRI focused on resorption characteristics and primary stability of the three-component anchor material PLGA/β-TCP/CS. Therefore, we did not include subjective clinical data. Furthermore, patient data collected earlier than 12 months were excluded to obtain a realistic picture of the degradation process of PLGA/β-TCP/CS within the relevant time frame between 18 and 21 months [8]. The study was limited to 48 patients with 82 suture anchors. Nevertheless, the data presented here strongly support the view that PLGA/β-TCP/CS is a very suitable anchor material in arthroscopic RCR. The study is limited by the relatively small number of included patients, and we did not directly compare this material with other anchor materials, although it seems that Regenesorb® suture anchors have superior resorption characteristics compared to commonly used and thus far reported bioabsorbable materials.

## Conclusion

The present study shows that PLGA/β-TCP/CS is a suitable biodegradable anchor material in arthroscopic RCR. The degradation process seems to be completed within 21 months, and the biomechanical characteristics are superior to those of the commonly used bioabsorbable anchor materials. There was no severe osteolysis or cyst formation (grades 3 and 4) observed around any of the 82 anchors. Furthermore, PLGA/β-TCP/CS showed sufficient primary stability for the 21-month observation period and led to the complete healing of the refixated supraspinatus tendon in 46 out of 48 (96%) patients. In our opinion, the use of PLGA/β-TCP/CS suture anchors in rotator cuff repair is a safe option for RCR and provides a superb bone stock in the case of revision surgery.

## Abbreviations

CS: Calcium sulfate
HA: Hydroxyapatite
MRI: Magnetic resonance imaging
PDLDA: Poly (dl-lactide)
PEEK: Polyetheretherketone
PLGA: Poly (lactide-co-glycolide)
PLLA: Poly (l-lactide)
RCR: Rotator cuff repair
β-TCP: Beta tricalcium phosphate
